# Mentalization and Emotion Regulation in Adolescent Attachment: A Scoping Review

**DOI:** 10.3390/children13030420

**Published:** 2026-03-19

**Authors:** Varvara Salavou, Katerina Papanikolaou, Artemios Pehlivanidis, Georgios Giannakopoulos

**Affiliations:** 1 Department of Child and Adolescent Psychiatry, School of Medicine, National and Kapodistrian University of Athens, Aghia Sophia Children’s Hospital, 11527 Athens, Greece; varvsalavou@med.uoa.gr (V.S.); katpapan@med.uoa.gr (K.P.); 2 1st Department of Psychiatry, National and Kapodistrian University of Athens, Eginition Hospital, 11528 Athens, Greece; apechlib@med.uoa.gr

**Keywords:** attachment, adolescence, mentalization, emotion regulation, epistemic trust, internalizing and externalizing symptoms, developmental psychopathology

## Abstract

**Highlights:**

**What are the main findings?**

- Across studies (2015–2025), adolescent attachment security was consistently linked to better emotion regulation and higher mentalization capacities, while emotion dysregulation was robustly associated with internalizing and externalizing psychopathology.
- Mentalization commonly operated as a pathway connecting attachment security to emotional outcomes (often as a mediator), and epistemic trust emerged as a related interpersonal mechanism associated with more adaptive functioning.

**What are the implications of the main findings?**

- Prevention and intervention efforts in adolescence may benefit from integrated targets—strengthening attachment-related security, mentalization/reflective functioning, and emotion regulation skills (and, where relevant, addressing maladaptive forms such as hypermentalizing).
- The field needs theoretically unified, multimethod and multi-informant assessment frameworks (beyond predominant self-report approaches) to improve comparability across studies and clarify patterns of vulnerability and resilience.

**Abstract:**

**Background**: Adolescence is a critical developmental period marked by reorganization of attachment relationships, heightened emotional reactivity, and ongoing maturation of reflective and regulatory capacities. Within this context, mentalization and emotion regulation have emerged as key concurrent associations linking attachment security to both vulnerability and resilience. This scoping review examined how mentalization and emotion regulation are conceptualized and operationalized in adolescent attachment research and synthesized empirical evidence on their interaction across clinical and non-clinical samples. **Methods**: Following PRISMA-ScR guidelines, four electronic databases were searched for peer-reviewed studies published between 2015 and 2025. Twelve studies met the inclusion criteria, encompassing community, high-risk, and clinical samples and employing cross-sectional, psychometric, and prospective designs. **Results**: Across studies, attachment security was consistently associated with more adaptive emotion regulation and higher mentalization capacities, whereas emotion regulation difficulties were linked to internalizing and externalizing psychopathology. Mentalization was frequently reported as a mediator or correlate in the association between attachment security and emotional outcomes, while epistemic trust emerged as a promising interpersonal concurrent association with adaptive functioning. **Conclusions**: Findings highlight differentiated patterns of vulnerability and resilience and underscore the need for theoretically integrated, multimethod assessment frameworks to guide future research and prevention efforts in adolescence.

## 1. Introduction

### 1.1. Attachment, Emotion Regulation and Mentalization in Adolescence

Adolescence represents a critical period of development, wherein attachment undergoes significant changes as individuals navigate emerging neurobiological, emotional, cognitive and social challenges [1,2]. Attachment in adolescence is of particular interest as it involves the reorganization of attachment patterns; individuals navigate higher levels of autonomy from parents and more emotional reliance on peers and romantic partners alongside ongoing negotiation of attachment needs with caregivers [3,4,5,6]. The re-appraisal of internal working models and the development of meta-cognitive and reflective capacities in the context of heightened emotional turbulence turn adolescence into a developmental phase of particular vulnerability and potential [7,8].

In the context of attachment, emotion regulation refers to intrinsic and extrinsic processes that influence how individuals respond to distress in accordance with situational and longer-term goals, particularly through the availability of attachment figures for comfort [9,10,11,12,13]. In adolescence, emotion regulation strategies become crucial both for interpersonal relationships and psychosocial adjustment [6,14,15].

Emotion dysregulation is more readily quantified than adaptive emotion regulation, as it interferes with goal-directed behavior and remains a broad risk factor for psychopathology [16]. Evidence shows that neural substrates interact with dysfunction in other neurobiological systems to produce maladjustment and, in clinical cases, vulnerability to internalizing and externalizing problems [17]. Emotion regulation difficulties in children and adolescents are associated with internalizing and externalizing problems and linked with impairment over and above specific disorders [18,19].

On the other hand, mentalization has been defined as the preconscious capacity to understand one’s own and others’ mental states in terms of emotions, wishes, desires, thoughts, and beliefs as well as intentions [20]. As a developmental achievement, mentalizing reflects increasing awareness of the importance of mental states for both intrapsychic and interpersonal processes [21].

In adolescence, mentalizing skills become particularly important as they have a strong impact on social relatedness, potential for intimacy in romantic relationships and group belonging. Compromised mentalizing skills have been associated with heightened risk for psychopathology [22]. In addition to mentalization, recent research has emphasized epistemic trust, defined as openness to the personal relevance and credibility of socially transmitted knowledge [23]. In empirical research, mentalization is often examined through reflective functioning measures, whereas epistemic trust refers to a distinct interpersonal process related to openness to social learning.

### 1.2. Mentalization and Emotion Regulation in Attachment

The relationship between mentalizing and emotion regulation has been described as bidirectional in childhood, wherein mirroring and emotional attunement with the mentalizing caregiver provide a social learning environment that gradually supports the development of autonomous emotion regulation [24]. Several other factors linked with temperament, heredity, family context and sociocultural environment, beyond dyadic relationships, have been since identified as significant for mentalization development [25].

Mentalizing is conceptualized as an umbrella construct integrating emotional awareness and cognitive perspective-taking, as well as self- and other-focused processes, and is closely linked to the attachment system in supporting emotion regulation at both behavioral and neural levels [25]. In adolescents with borderline features, mentalizing failures and affect dysregulation appear to reinforce one another, forming a self-perpetuating cycle [26], while cumulative childhood trauma has been associated with severe PTSD in association with hypomentalizing and maladaptive emotion regulation [27].

Despite growing empirical interest, research on adolescent attachment, mentalization, and emotion regulation remains theoretically dispersed and methodologically heterogeneous. Studies vary in how these constructs are conceptualized and measured, limiting cross-study comparability and making it difficult to synthesize evidence within adolescent attachment research. A synthesis of how mentalization and emotion regulation are examined together in adolescence is currently lacking.

### 1.3. Objectives

Evidence for the mediating role of emotion dysregulation in the mentalizing of adolescents with borderline features [28] supports the notion of an association between mentalization and emotion regulation. Evidence also suggests that increased mentalizing may reduce children’s emotional stress responses at the psychophysiological level [29]. However, a comprehensive synthesis of the developmental phase of adolescence remains lacking.

Given the clinical relevance of both constructs and their association with developmental outcomes, we conducted a scoping review to synthesize evidence in the field of their interplay over the last decade (2015–2025) with community, clinical and high-risk (e.g., residential care settings) samples. Our main aim was to identify, categorize and synthesize findings from existing studies. In particular, we aimed: (a) To examine the conceptualization and operationalization of constructs such as mentalization and emotion regulation in adolescent attachment and (b) To map existing evidence from studies exploring the association between emotion regulation and mentalization in the developmental phase of adolescence.

## 2. Materials and Methods

### 2.1. Protocol and Registration

Scoping reviews are a useful method to synthesize findings when the topic has never been examined in depth. This method was also used to identify key concepts and analyze knowledge gaps [30]. The protocol for this scoping review was registered on the Open Science Framework after completion of the screening process (doi: 10.17605/OSF.IO/ZX736). The review question, eligibility criteria, and general methodological approach had been established before screening began; however, formal registration was completed retrospectively rather than prospectively. This should be considered a limitation in procedural transparency. PRISMA Extension for Scoping Reviews (PRISMA-ScR) guidelines, a reporting guide for authors designed to increase the transparency and completeness of reporting methods for scoping reviews, were followed and fulfilled [31].

### 2.2. Eligibility Criteria

Studies were included in this review based on the following criteria: (1) they were written in English; (2) were published in peer-reviewed journals; (3) reported measurements related to emotion regulation and mentalization (including reflective functioning indices and performance-based mentalizing tasks); (4) were empirical studies (only quantitative, qualitative and mixed-method studies were included); (5) were carried out with adolescent samples. Restrictions were placed on the year of the publication, including only studies between 2015 and 2025.

### 2.3. Search Strategy

**Figure 1:**
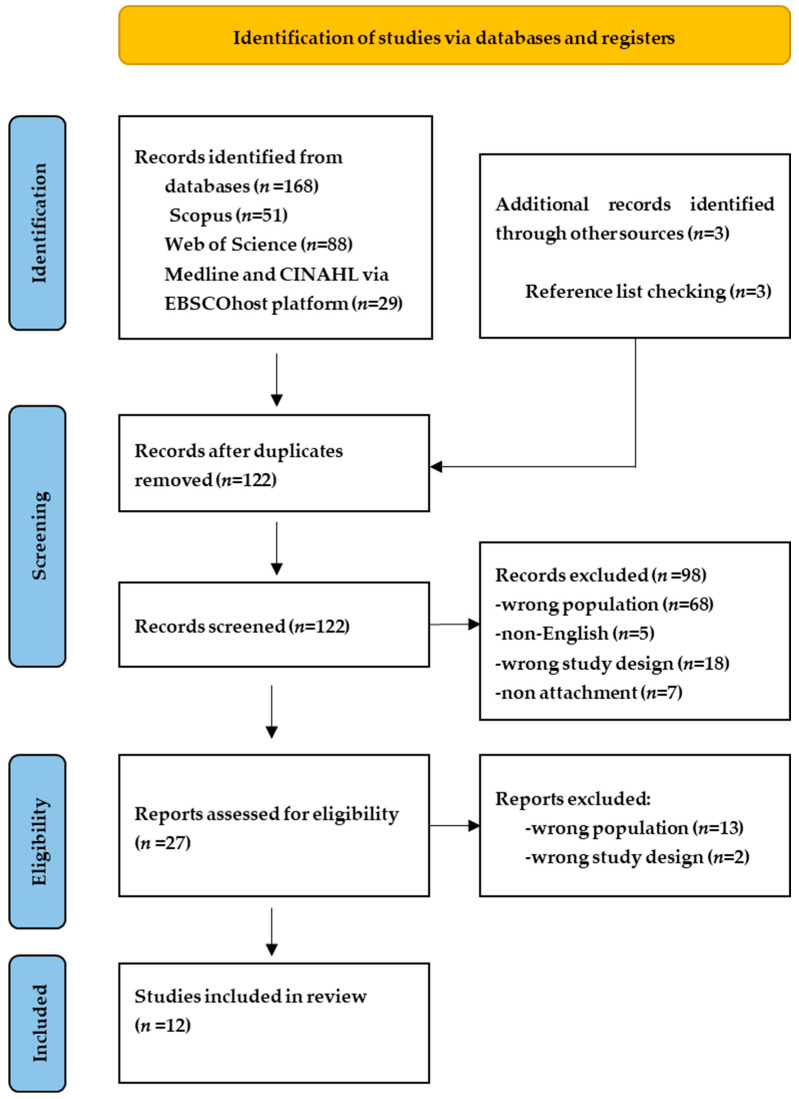
PRISMA flow diagram for the study selection process.

The search was performed across four databases (Scopus, Web of Science, MEDLINE and CINAHL via the EBSCOhost platform on NKUA) on 24 December 2025. The databases were selected to provide broad coverage of multidisciplinary, biomedical, nursing/allied health, and citation-indexed literature relevant to the review topic. Scopus and Web of Science were used for broad interdisciplinary coverage, while MEDLINE and CINAHL were included to capture medical, developmental, and mental health-related studies relevant to adolescent attachment, emotion regulation, and mentalization. We acknowledge, however, that the exclusion of PsycINFO may have limited coverage of some psychologically oriented studies, and this should be considered when interpreting the completeness of the evidence base. The search string was created with the Boolean operators AND/OR, nested with the keywords identified, and restricted to title, abstract and keywords. The search string chosen was ‘attachment’ AND (emotion regulation OR dysregulation) AND (mentalization OR reflective function), with further filters applied on years (2015–2025) and peer-reviewed journal publications. Full-text accessibility was required at the screening stage to allow eligibility assessment. The database search was performed by GG and VS. All studies retrieved from the complete search were exported to the systematic review web application Rayyan (https://rayyan.qcri.org, accessed on 24 December 2025). Each part of the selection process was performed by VS, and uncertainties or borderline cases were resolved through discussion with GG. The complete screening process is detailed in the PRISMA flow diagram (Figure 1).

### 2.4. Data Extraction

The data extraction was conducted by the first author (VS). The data collected from full-text articles were general study information (title, author, year, country, aim), methodology (sample characteristics and study design), measures related to emotion regulation and mentalization, and key findings.

### 2.5. Quality Assessment

Although formal critical appraisal is not mandatory in scoping reviews, a basic indication of the methodological strength of the included studies is useful for interpretation. Therefore, we conducted a descriptive quality appraisal of the 12 included studies to contextualize the evidence base, without excluding studies on the basis of quality. Given the heterogeneity of designs, the appraisal focused on broad methodological features relevant to evidential strength, including study design, sample characteristics, inclusion of clinical or high-risk groups, reliance on self-report versus interview-, narrative-, or performance-based methods, use of validated measures, and the presence of longitudinal, comparative, or multi-informant data. The results of this appraisal are summarized in Table S3 and integrated narratively into the synthesis and discussion.

### 2.6. Synthesis of Results

First, studies were categorized by year of publication, country of investigation, population characteristics, type of study design and key findings. We organized key findings according to the topics investigated (emotion regulation or dysregulation and mentalization or reflective function) and checked thoroughly for repeated patterns and new evidence, according to existing literature. Then we created a second table recording the specific measures used on attachment, emotion regulation and mentalization in an attempt to map the conceptualization and operationalization of the constructs within existing studies from the last decade.

## 3. Results

### 3.1. Study Selection

The complete search led to the identification of 168 articles. After duplicates were removed (*n* = 46), a total of 122 articles were screened based on titles and abstracts against eligibility criteria. The reasons for exclusion included: (1) wrong population (studies that did not assess adolescents (*n* = 68)); (2) non-English language (*n* = 5); (3) wrong study design (non-empirical studies (*n* = 18)); (4) non-attachment (outside attachment context (*n* = 7)). In addition, three studies meeting the inclusion criteria were identified through reference list checking and were included in the review.

Of the 27 full-text articles that were screened, 15 were excluded (13 were excluded due to the wrong population and 2 due to the wrong study design). Thus, a total of 12 studies were finally included in the review. The complete record selection process is summarized in the PRISMA flow diagram (Figure 1).

### 3.2. General Characteristics of the Included Studies

Twelve studies met the eligibility criteria for the review. Two included papers reported separate psychometric studies. These were synthesized independently due to differences in sample and psychometric analyses. A full description of study characteristics can be viewed in Table S1, while the measures used across studies to operationalize attachment, mentalization, emotion regulation, and epistemic trust are summarized in Table S2.

### 3.3. Countries and Publication Dates

Most studies were conducted in Italy (*n* = 6), followed by the UK (*n* = 3), Poland (*n* = 2), and the USA (*n* = 1). A total of 76.9% of the studies were conducted between 2021 and 2025, and 58.3% were conducted online, especially given the COVID-19 pandemic context.

### 3.4. Sample Characteristics

Overall, the twelve studies that met eligibility criteria for this scoping review referred to predominantly Western/European samples. Across studies, adolescent age ranged from 10 to 19 years; where reported, % female ranged from 44.0% to 100%. The most common study design was cross-sectional (*n* = 6), followed by psychometric validation studies (*n* = 4). One prospective observational cohort study (*n* = 1) and one case–control analytic study (*n* = 1) also reached eligibility criteria. The majority of studies in this review used community samples (*n* = 6), followed by clinical samples (*n* = 3) and mixed samples with low and high-risk adolescents (*n* = 3).

### 3.5. Overview of Methodological Strength of Included Studies

A basic appraisal of the included studies indicated that the evidence base is informative but methodologically uneven (Table S3). Most studies were cross-sectional (*n* = 6), with only one prospective observational cohort study and one case–control analytic study, limiting causal inference across much of the literature. Four studies were psychometric validation studies, which strengthened understanding of measurement properties but did not directly address developmental or causal pathways. Community samples predominated (*n* = 6), while only three studies included exclusively clinical samples and three used mixed or high-risk samples, restricting the clinical generalizability of the overall synthesis. In terms of measurement, adolescent self-report instruments predominated, particularly for mentalization and emotion regulation, including repeated use of RFQ-based measures and the DERS. Only a smaller subset of studies incorporated semi-structured interviews, narrative methods, or performance-based approaches, such as the FFI, CAI, MASC, MST, or ASSP, which broadened construct coverage beyond self-report alone. One study included parental data and thus offered a multi-informant perspective, but most studies relied on adolescent reports alone. Overall, the evidence is best interpreted as preliminary to moderate in strength: recurring associations across studies are informative, but conclusions regarding causality, developmental directionality, and the measurement of mentalization in clinical populations should remain cautious.

### 3.6. Results of Individual Sources of Evidence

Table S1 provides a summary of all included studies, including their year, country, aim, sample characteristics, study design and key findings relevant to the review objectives. Across the included studies, attachment security, mentalization, emotion regulation, and epistemic trust were examined in both community and clinical adolescent samples using cross-sectional, psychometric, and prospective designs. In community samples, secure attachment to parents—particularly to fathers—was frequently associated with fewer difficulties in emotion regulation, while mentalization abilities were positively related to adaptive regulatory strategies and psychological resources. Some studies reported that mentalization partially mediated the association between attachment security and emotion regulation, whereas attachment to fathers emerged more frequently than attachment to mothers as a predictor of adolescents’ mentalizing abilities within the included studies. Emotion dysregulation was commonly linked to internalizing and externalizing difficulties, including stress, binge-eating risk, and behavioral problems.

Psychometric studies generally demonstrated stable factorial structures and adequate to excellent internal consistency for instruments assessing mentalized affectivity and epistemic trust in adolescent samples, alongside meaningful associations with mentalization, emotion regulation, self-efficacy, and psychopathology. In contrast, a brief self-report measure of reflective functioning showed poor structural validity and limited sensitivity to change in a clinical adolescent sample with restrictive eating disorders. Studies examining epistemic trust indicated that higher epistemic trust was associated with greater mentalization and adaptive functioning, whereas epistemic mistrust and credulity were related to increased emotion dysregulation and internalizing symptoms. Clinical and high-risk samples—including adolescents with anorexia nervosa, borderline features, or residential-care histories—showed higher rates of insecure or disorganized attachment, lower narrative coherence and reflective functioning, and more pronounced emotion regulation difficulties compared to community peers. Prospective findings further indicated that parental mentalization and early therapeutic alliance were more strongly associated with treatment outcomes than attachment classifications alone.

Table S2 summarizes the measures used across the twelve studies to operationalize attachment, mentalization, emotion regulation, and epistemic trust. Across studies, these constructs were assessed through validated self-report, interview-based, and performance-based instruments. Attachment was operationalized using the Security Scale [32], the Attachment Style Questionnaire (ASQ) [33], the Inventory of Parent and Peer Attachment (IPPA) [34], the Experiences in Close Relationships Scale (ECRS) [35], the Child Attachment Interview (CAI) [36], the Friends and Family Interview (FFI) [37], and the Adolescent Story Stem Profile (ASSP) [38]. Emotion regulation was primarily assessed with the Difficulties in Emotion Regulation Scale (DERS) [39] and the Toronto Alexithymia Scale (TAS-20) [40]. Mentalization and related constructs were measured using the Reflective Functioning Questionnaire (RFQ) [41], the Reflective Functioning Questionnaire for Youth (RFQ-Y) [42], the five-item version (RFQ-Y-5) [43], the Epistemic Trust, Mistrust and Credulity Questionnaire (ETMCQ) [44], the Mentalized Affectivity Scale for Adolescents (B-MAS-A) [45], the Mental States Task (MST) [46], and the Movie for the Assessment of Social Cognition (MASC) [47].

Overall, self-report measures predominated, particularly for mentalization and emotion regulation, whereas interview and narrative-based approaches captured attachment and mentalization within relational contexts. This heterogeneity in measurement approaches reflects differences in theoretical assumptions regarding the trait-like versus context-sensitive nature of the constructs.

### 3.7. Synthesis of Results

Given the limited number of studies meeting the eligibility criteria, findings were synthesized across three domains. Results are presented with emphasis on how individual studies contribute to the review objectives, rather than on detailed reporting of study-specific statistical indices. The synthesis focused on identifying recurring associations across studies while noting differences in samples, measurement approaches, and study designs.

#### 3.7.1. Psychometric Evidence in Adolescent Samples

Overall, four studies examined the psychometric properties of measures assessing attachment, mentalization, affective competency, mentalized affectivity, and epistemic trust in adolescent samples (Table S2). In a clinical cohort of adolescents with restrictive eating disorders, Jewell et al. [48] reported that the five-item version of the Reflective Function Questionnaire for Youth (RFQY-5) showed poor structural validity, low internal consistency, partial convergent validity, and limited sensitivity to change over nine months, indicating limited suitability for this population.

In contrast, psychometric support was stronger for multidimensional measures in community samples. Liotti et al. [45] demonstrated that the Brief-Mentalized Affectivity Scale for Adolescents (B-MAS-A) exhibited a stable three-factor structure (identifying, processing, and expressing emotions) with excellent internal consistency. Significant age- and gender-related differences emerged across dimensions, indicating developmental and gender-specific variation in mentalized affectivity. Similarly, Milesi et al. [49] confirmed the original three-factor structure of the Epistemic Trust, Mistrust and Credulity Questionnaire (ETMCQ), with adequate internal consistency and partial measurement invariance across gender; age was weakly and positively associated only with epistemic trust.

Preliminary findings on the Adolescent Story Stem Profile (ASSP) by Zhang et al. [38] supported a three-factor structure capturing story-self relevance, attachment, and mentalization/affect competency, accounting for 52% of the variance. Internal consistency and cross-scale correlations were satisfactory; however, ASSP subscales were not significantly associated with the RFQY-5, suggesting potential differences between narrative-based and self-report measures of mentalizing. No group differences were observed on the attachment dimension.

Across the psychometric studies, psychometric evidence was mixed. Multidimensional measures showed stronger support in community samples, whereas one study reported poorer structural validity for a brief reflective functioning measure in a clinical population.

#### 3.7.2. Attachment, Mentalization, and Emotion Regulation

Evidence across studies indicated consistent associations between attachment security, mentalization, and emotion regulation [45,50,51]. Liotti et al. [45] showed that higher mentalized affectivity was associated with lower alexithymia, greater certainty about mental states, and higher epistemic trust, supporting the construct and criterion validity of the B-MAS-A. Similarly, Milesi et al. [49] reported that epistemic trust was positively associated with reflective functioning and attachment-related trust toward parents and peers, whereas epistemic credulity was linked to greater emotion dysregulation and lower relational trust.

Narrative-based findings further highlighted heterogeneity in mentalization and affective functioning across risk groups. Using the ASSP, Zhang et al. [38] found no group differences in overall mentalizing abilities between low- and high-risk adolescents, although low-risk adolescents showed higher frequencies of positive mentalizing, while high-risk adolescents demonstrated greater accuracy in emotion recognition and higher affective competence scores.

In nonclinical samples, attachment anxiety was consistently associated with greater emotion dysregulation, whereas attachment avoidance showed weaker or more specific associations [51]. Mentalization demonstrated modest but significant links with emotion regulation, particularly in relation to emotional clarity and nonacceptance. Attachment anxiety and mentalization jointly predicted emotion dysregulation, with attachment anxiety accounting for a larger proportion of variance.

Epistemic trust was positively associated with reflective functioning, whereas epistemic mistrust and credulity were associated with greater difficulties in emotion regulation [52]. In the same study, reflective functioning showed a weak but significant negative association with emotion regulation difficulties. Gambin et al. [50] further showed that attachment security to both parents predicted fewer emotion regulation difficulties, while attachment security to fathers uniquely predicted mentalizing abilities. Mentalization partially mediated the association between father attachment security and emotion regulation difficulties, with stronger effects observed in younger adolescents.

Additional evidence linked attachment insecurity to maladaptive outcomes in specific contexts. Pace et al. [53] found that adolescent females at risk for binge-eating behavior showed higher rates of insecure attachment, greater anger toward mothers, and poorer attachment-related functioning, with both dismissing and preoccupied attachment patterns predicting binge-eating severity. During the COVID-19 lockdown, Locati et al. [54] identified mentalization and epistemic trust as protective factors, with differentiated effects by attachment figure: trust in fathers was associated with lower perceived stress, whereas trust in mothers was associated with fewer emotion regulation difficulties.

In clinical samples, Sharp et al. [55] reported that higher attachment security was associated with lower hypermentalizing, while both hypermentalizing and emotion dysregulation were linked to borderline features. Mediation analyses indicated that hypermentalizing, but not emotion dysregulation alone, functioned as an independent mediator between attachment security and borderline features. In adolescents with anorexia nervosa, Jewell et al. [56] found that attachment security was associated with stronger therapeutic alliance and that parental hypermentalizing predicted poorer treatment outcomes, suggesting that mentalization may influence outcomes indirectly through alliance formation.

Across studies, attachment security was generally associated with more adaptive emotion regulation and higher mentalizing capacities, whereas attachment anxiety showed more consistent associations with emotion dysregulation than attachment avoidance (Table S1). At the same time, findings varied across samples and measurement approaches, particularly between community and clinical samples and between self-report and narrative or performance-based assessments of mentalization. The majority of included studies relied on self-report measures to assess mentalization and emotion regulation (Table S2).

#### 3.7.3. Mentalization, Emotion Dysregulation, and Psychopathology

Across studies, emotion dysregulation emerged as a central correlate of internalizing and externalizing difficulties [45,49,52,55] (Table S1). Liotti et al. [45] found that higher mentalized affectivity was associated with fewer behavioral and emotional problems, greater self-efficacy, and higher prosocial behavior. In contrast, Milesi et al. [49] showed that epistemic mistrust and credulity were positively associated with emotion dysregulation and psychopathology, whereas epistemic trust was not directly related to symptom severity.

Parolin et al. [52] further demonstrated that epistemic mistrust and credulity were associated with more serious internalizing problems both directly and indirectly through reduced mentalization and increased emotion dysregulation, whereas reflective functioning was not directly associated with internalizing symptoms. Sharp et al. [55] reported no gender differences in internalizing or externalizing symptoms, despite higher borderline features in females, suggesting that symptom severity patterns may not map straightforwardly onto gender differences in related personality features.

Finally, longitudinal findings by Jewell et al. [56] indicated that parental over-certainty about mental states—as an indicator of hypermentalizing—was associated with poorer treatment outcomes in adolescents with anorexia nervosa. Adolescent emotion regulation difficulties showed a more complex pattern, as only higher levels of lack of emotional clarity were associated with increased odds of recovery. Difficulties in emotional awareness were linked to lower therapeutic alliance, and alliance ratings in turn predicted positive outcomes, suggesting an indirect pathway through early relational processes. Higher reflective functioning was associated with stronger therapeutic alliance, underscoring the relevance of mentalization processes for alliance formation in clinical contexts.

Across studies, emotion dysregulation emerged as a consistent correlate of psychopathology. Moreover, mentalization-related processes—such as reflective functioning and epistemic trust—showed more complex and context-dependent associations, depending on the measures used and the clinical characteristics of the samples.

## 4. Discussion

This scoping review aimed to examine the conceptualization and operationalization of mentalization and emotion regulation in adolescent attachment. It also sought to identify recurring patterns and gaps across studies exploring their interplay.

### 4.1. Toward an Integrated Framework

Within the twelve included studies, mentalization was examined across community, high-risk, and clinical adolescent samples, primarily in relation to emotion regulation and internalizing–externalizing difficulties. Across studies, mentalization and emotion regulation were operationalized through partially overlapping yet theoretically non-integrated constructs, limiting conceptual coherence and comparability. This observation suggests that the field currently lacks an integrated developmental framework linking attachment, mentalization, and emotion regulation in adolescence.

Mentalization was predominantly assessed via self-report reflective functioning indices [48,50,54] and RFQ-based variants used for convergent validity [38,45,49,52], reflecting a largely trait-oriented operationalization. Fewer studies employed performance-based tasks [38,49] or narrative interviews [52,53], capturing mentalization as context-sensitive and relationally embedded. This variability reflects an ongoing conceptual tension regarding whether adolescent mentalization is best understood as a stable trait, a context-dependent capacity, or a relational process.

Emotion regulation difficulties were similarly assessed primarily through a self-report measure (DERS) emphasizing intrapersonal regulatory capacities [48,49,50,51,52,54,55,56]. By contrast, narrative-based assessment approaches—including a semi-structured interview [53,57] and a structured story-stem task [38]—situated regulation within attachment-related contexts. A similar methodological distinction thus emerges in the assessment of emotion regulation, with self-report measures predominating. Although the DERS has demonstrated validity in adolescent and clinical populations [58], its emphasis on intrapersonal regulatory difficulties may partly explain the strong and consistent association reported between emotion dysregulation and psychopathology across studies.

Epistemic trust emerged in a limited but growing subset of community studies [45,49,54], extending mentalization into a broader interpersonal domain and linking it with adaptive emotional functioning. Mentalized affectivity, as described by Jurist [59], examined in one validation study [45], represents an integrative attempt to bridge mentalization and emotion regulation within a unified construct. Both constructs may be especially salient in adolescence—a developmental phase characterized by heightened emotional intensity, expanding reflective capacities, increasing reliance on attachment figures beyond the family context, and still fragile regulatory processes.

The proliferation of RFQY variants reflects ongoing conceptual ambiguity [60]. This issue is particularly important for interpreting findings in clinical adolescent samples. The poor structural validity, low internal consistency, partial convergent validity, and weak sensitivity to change observed for the RFQY-5 in adolescents with restrictive eating disorders [48] raise a broader measurement question for the field.

Brief self-report RFQ-based indices may not always capture a stable latent mentalization construct under conditions of high psychopathology, intense affective arousal, or rigid self-representational styles. In such contexts, scores may reflect a mixture of genuine reflective functioning and state-dependent distress. They may also capture defensive certainty or uncertainty about mental states, reading-comprehension demands, or general self-evaluative bias rather than mentalization per se.

Accordingly, some of the inconsistencies in clinical findings may reflect measurement noise rather than the true absence or presence of mentalizing capacity. This does not invalidate the construct of mentalization itself, but it does suggest caution in treating RFQ-based scores—especially ultra-brief versions—as straightforward proxies of stable reflective functioning in clinical populations. A more defensible approach for future research would be to triangulate self-report data with interview-based, narrative, observational, and performance-based indicators. This would help distinguish trait-like reflective capacity from context-sensitive fluctuations and instrument-specific error.

Meta-analytic evidence supports the use of attachment interviews in adolescence [61,62], which allow for multidimensional assessment of relational emotion regulation and mentalization processes beyond attachment classification. Differences in effect sizes between questionnaire and representational measures [63] further complicate integration across studies. In addition, adolescent self-report remains vulnerable to recall and salience biases [64], which may inflate associations and obscure developmental nuance. With the exception of one clinical study including parental measures [56], the majority of included studies relied exclusively on adolescent self-report data. Evidence supports that single-informant data may inflate associations and that informant discrepancies may reflect meaningful attachment and mentalization-related processes rather than mere measurement error [65,66].

Overall, while some studies focus on the interplay between attachment and emotion regulation, others examine mentalization in relation to psychopathology or explore potential mediating processes within broader developmental patterns. Across community, high-risk, and clinical samples, attachment, mentalization, and emotion regulation are operationalized within partially overlapping frameworks. This conceptual fragmentation limits comparability across studies.

In addition, the descriptive appraisal of included studies suggests that the field is constrained by a predominance of cross-sectional and self-report designs, with relatively limited clinical, longitudinal, and multi-informant evidence. Future research would benefit from multimethod and multi-informant designs grounded in unified theoretical models capable of capturing both state-dependent and trait-like aspects of socio-emotional functioning. This is especially important in clinical populations, where findings from a recent validation study [48] leave open the possibility that some self-report ‘mentalization’ scores reflect substantial measurement noise rather than a stable underlying construct. Clarifying whether mentalization and emotion regulation represent partially overlapping constructs or hierarchically organized processes—and how best to measure them under psychopathological conditions—remains a central theoretical and methodological challenge.

### 4.2. Vulnerability and Resilience

Within the limited evidence from three included studies [50,51,54], attachment security to mothers appeared to be associated with fewer difficulties in emotion regulation, whereas attachment security to fathers was more indirectly related to resilience in relation to adolescents’ mentalizing capacities. These findings should be interpreted cautiously, given the small number of studies and the possibility that this pattern may reflect developmental dynamics specific to adolescence—when paternal relationships may become more salient for autonomy and social-cognitive development. Measurement-related factors may also contribute to this finding, as both attachment and mentalization were assessed using different instruments across the three studies, including self-report questionnaires and task-based assessments of mentalizing. Limited evidence from a late adolescent female sample [51] suggests that attachment anxiety and reduced mentalizing capacity may confer vulnerability within romantic relationship contexts, which may be characterized by heightened affective arousal. However, this finding derives from a female-only sample, and future research should examine whether similar patterns emerge among male late adolescents.

Evidence in the present review underscores the importance of caregiving stability in supporting attachment security during adolescence. However, findings comparing high-risk and low-risk groups in reflective functioning and emotion regulation were mixed, pointing to differentiated patterns of vulnerability and resilience. While one study reported lower mentalization and greater affect regulation difficulties among high-risk adolescents [57], another found no significant group differences and even higher affective competence in high-risk samples [38]. These contrasting findings may indicate that exposure to adversity does not uniformly result in impaired functioning; rather, mentalization and affect regulation may operate as vulnerability or resilience-related processes depending on contextual and developmental conditions. Such heterogeneity likely reflects differences in sample characteristics, operationalization, and assessment methods, highlighting the need for further research across diverse risk contexts.

Within the included studies [45,49,54], higher epistemic trust was associated with greater mentalization capacities and more adaptive emotional functioning, whereas epistemic mistrust and credulity were linked to dysregulation and internalizing vulnerability. Although epistemic trust emerges as a promising construct, evidence remains preliminary and unevenly distributed across attachment figures and samples.

Across the included studies, emotion regulation difficulties consistently emerged as a transdiagnostic mechanism implicated in borderline features, eating pathology, and internalizing–externalizing symptoms [53,55,56]. This pattern may be understood within transdiagnostic conceptualizations of psychopathology [18,67].

Importantly, limited evidence suggests that both hypermentalizing and emotion regulation difficulties may jointly mediate the association between attachment and psychopathology, particularly borderline features, when these processes are examined together [55]. In this context, hypermentalizing appears to represent the primary mediating mechanism, while emotion regulation difficulties may contribute as part of a broader socio-emotional pathway.

Although intervention research outside the present review suggests potential benefits of targeting parental emotion regulation and mentalization [68,69], evidence within this scoping review remains limited to one study suggesting that parental hypermentalizing may be a risk factor for poorer treatment outcomes in anorexia nervosa [56]. Parental mentalizing may represent an underexplored pathway in adolescent vulnerability and resilience. From a clinical perspective, mentalization-based approaches and emotion regulation-focused programs may therefore represent complementary targets for preventive and therapeutic interventions for adolescents. Strengthening reflective functioning and regulatory capacities within attachment relationships may help reduce vulnerability to internalizing and externalizing difficulties [68], highlighting the potential relevance of transactional and intergenerational processes in intervention research [69].

Although the number of included studies is limited, the findings suggest that mentalization and emotion regulation consistently emerge as central processes associated with vulnerability and resilience across community, high-risk, and clinical samples. Epistemic trust appears to extend these processes into broader interpersonal domains.

The heterogeneity observed across samples and methods underscores the developmental sensitivity of these constructs. Clarifying developmental patterns to psychopathology and resilience remains a central priority for future research, particularly in studies examining adolescent and parental mentalization and emotion regulation.

### 4.3. Limitations

Several limitations should be acknowledged. First, although we included a basic descriptive appraisal to contextualize the included studies, we did not conduct a full formal risk-of-bias assessment using design-specific appraisal tools. Accordingly, the strength of the reported associations should be interpreted cautiously, particularly where findings derive from cross-sectional designs, predominantly self-report measures, or relatively small clinical or high-risk subgroups. The present review is therefore better suited to mapping patterns, conceptual tendencies, and methodological gaps in the literature than to drawing firm conclusions about the magnitude or causal direction of effects. In addition, the protocol was registered retrospectively, after completion of the screening process, rather than prospectively, which reduced procedural transparency. Second, the relatively small number of included studies (*n* = 12) limits the strength and generalizability of the conclusions, and findings should therefore be interpreted with caution.

Third, six studies were cross-sectional (excluding four psychometric validation studies), limiting causal inference between attachment, mentalization, and emotion regulation processes. Fourth, only a small number of studies (*n* = 3) included clinical or high-risk adolescent samples, restricting the generalizability of findings to more vulnerable populations and limiting conclusions regarding psychopathology-related outcomes. Fifth, the predominance of Western, and particularly Southern European, samples limits the cultural generalizability of the findings, especially given cultural variation in attachment-related practices, emotion socialization, and mentalization processes. These cultural differences may influence how attachment relationships are expressed, how emotional experiences are regulated, and how reflective functioning is manifested across developmental contexts. As a result, the patterns identified in this review may not fully reflect attachment-related processes in non-Western or more diverse sociocultural settings. Sixth, the restriction to English-language publications may have introduced language bias and may have resulted in the exclusion of potentially relevant studies published in other languages.

In addition, although the search covered four major databases with a relevant interdisciplinary and health-related scope, the exclusion of PsycINFO may have reduced sensitivity to psychologically focused literature, particularly studies indexed primarily within psychology and psychotherapy databases. Finally, two included studies were derived from the same cohort. However, this reflects the reuse of data to address distinct research questions and analytic aims rather than duplication of findings, and both studies contributed unique information relevant to the scope of the review.

## 5. Conclusions

Overall, the findings of this scoping review highlight mentalization, mentalized affectivity, and epistemic trust as distinct yet complementary protective processes during adolescence. Epistemic trust appears to function as an interpersonal condition that facilitates mentalization, while mentalized affectivity operates as an intrapersonal mechanism supporting the integration and regulation of emotional experience. Together with broader emotion regulation capacities, these processes appear to be associated with patterns of vulnerability and resilience during adolescence, extending beyond attachment classifications and exposure to adverse childhood experiences.

Long-standing calls to examine differential associations from parent–child attachment to internalizing versus externalizing outcomes, as well as the role of mediating and moderating processes [70], are reflected in the gradual yet meaningful progress captured in this review. The accumulating evidence suggests that emotion regulation, mentalization, and epistemic trust represent key processes through which attachment-related factors influence development and developmental psychopathology. However, this evidence base remains methodologically uneven and should be interpreted in light of the predominance of cross-sectional, self-report, and community-based studies. At the same time, the present review indicates that the measurement of mentalization—particularly through brief self-report RFQ-based variants in clinical adolescent samples—remains insufficiently resolved, and future work must determine more clearly when such instruments capture stable reflective functioning and when they primarily index transient distress or measurement noise. In parallel, the findings may provide a conceptual basis for future research examining interventions targeting adolescents’ emotion regulation and mentalization capacities, as well as parental processes that support these skills [68,69,71,72,73].

Finally, mentalization and emotion regulation may be tentatively conceptualized as part of broader developmental cascades [74], with the potential to influence multiple domains of psychological functioning through dynamic interactions with relational and contextual systems over time. Advancing understanding of adaptive and maladaptive developmental patterns, and further examining how mentalization and emotion regulation are associated with adolescent functioning, remain central challenges for research and practice—particularly during adolescence, a period of heightened plasticity and opportunity for change.

## Data Availability

The original contributions presented in this study are included in the article and Supplementary Materials. Further inquiries can be directed to the corresponding author.

## Supplementary Materials

The following supporting information can be downloaded at: https://www.mdpi.com/article/10.3390/children13030420/s1, Table S1: Characteristics of the included studies; Table S2: Attachment, emotion regulation and mentalization measures of included studies; Table S3: Basic methodological appraisal of included studies.
