# Supplementary material for: Mentalization and Emotion Regulation in Adolescent Attachment: A Scoping Review
Source: Children (Basel). 2026 Mar 19;13(3):420. doi: 10.3390/children13030420 (PMC13025779; doi:10.3390/children13030420)
Supplement: Supplementary file 1 [file children-13-00420-s001.zip › children-4204267-supplementary.pdf]

Table S1. *Characteristics of the included studies*

Authors and Year	Country	Aim	Sample characteristics *N* (gender distribution) Age=range (*M, SD*)	Study design	Key findings
Gambin et al. (2021) [50]	Poland	To investigate the relations between attachment security to mother and father, mentalizing abilities and difficulties in the use of emotion regulation strategies in adolescents.	Community sample of adolescents recruited from high-schools<br><br>*N*=530 (286 females)<br><br>Age=15-18<br>*(M=16.82, SD=.71)*	Cross-sectional	Secure attachment to both mother and father was associated with fewer emotion regulation difficulties in adolescents.<br>Attachment to father, but not mother, significantly predicted adolescents' mentalizing abilities. Mentalizing partially mediated the relationship between father attachment security and emotion regulation.
Jewell et al. (2023) [56]	UK	To examine whether adolescent and parental attachment styles and mentalization predict treatment outcomes in family therapy for adolescent anorexia nervosa, and to explore the role of early therapeutic alliance.	Clinical sample: Adolescents with anorexia nervosa from three specialist community-based eating disorders services<br><br>Adolescents: *N*=173 (153 female) Parents: *N*=163 (149 mothers)<br><br>Age=11-17<br>*(M=14.69, SD=1.54)*	Prospective observational cohort study	Parental impaired mentalization, particularly over-certainty about mental states, predicted poorer treatment outcomes.<br>Stronger therapeutic alliance at one month was associated with better outcomes.<br>Attachment and mentalization were related to therapeutic alliance, but mentalization (especially parental) emerged as the most relevant predictor of outcome.

Authors and Year	Country	Aim	Sample characteristics *N* (gender distribution) Age=range (*M, SD*)	Study design	Key findings
Jewell et al. (2024) [48]	UK	To test a brief, psychometrically-sound self-report measure of adolescent reflective functioning in an eating disorder sample, the Reflective Function for Youth-5 item version.	Clinical sample: adolescents with a diagnosis of Anorexia Nervosa or restrictive Other Specified Feeding or Eating Disorder (OSFED) *N*=171 (151 females)<br><br>Age=10-17 *(M=14.7, SD=1.54)*	Psychometric validation study, conducted within a prospective observational cohort	Reflective Function Scale for Youth-5 item showed poor structural validity, low internal reliability, partial convergent validity, and was not sensitive to change over nine months in adolescents with restrictive eating disorders.
Liotti et al. (2023) [45]	Italy	To validate the Brief Mentalized Affectivity Scale for adolescents in the Italian population.	Community sample recruited from high schools	Psychometric validation study	
		Study 1: To check the psychometric properties of B-MAS-A, check for differences in gender and age among factors.	Study 1: *N*=566 (322 females)<br><br>Age= 13-19 *(M=16.59, SD=1.62)*	Study 1: Confirmatory factor analysis	Study 1: The B-MAS-A exhibited a three-factor structure (identifying, processing, expressing emotions) consistent with adult versions, with excellent internal consistency across subscales.

**Authors and Year**	**Country**	**Aim**	**Sample characteristics *N* (gender distribution) Age=range (*M, SD*)**	**Study design**	**Key findings**
Liotti et al. (2023) [45] *(continued)*	Italy	Study 2: To explore convergent validity with other mental functioning capacities such as mentalization, alexithymia, epistemic trust, levels of psychological resources and difficulties as well as perceived self-efficacy.	Study 2: *N*=288 (146 females)<br><br>Age=13-19 with divisions between early, middle and late adolescence *(M=15.92, SD=1.58)*	Study 2: Convergent validity testing	Study 2: All B-MAS-A subscales showed good construct and criterion validity, with meaningful associations — e.g., positive relations with epistemic trust and self-efficacy and negative relations with alexithymia and psychological difficulties — supporting the scale's validity in adolescents.
Locati et al. (2023) [54]	Italy	To investigate the relationship between mentalizing and stress (stress being the context of rapid onset of COVID-19 pandemic and lockdowns)<br><br>To explore whether mentalizing can be a protective factor in highly stressful circumstances and whether mentalizing can be undermined within highly stressful circumstances.	Community sample recruited from middle and high schools<br><br>*N*=131<br><br>Age=12-18 *(M=15.37, SD=1.85)*	Cross-sectional	Trust to mother, trust to father and trust to peers were all positively associated to one another and so were measures of stress and emotional dysregulation.<br><br>Epistemic trust towards father was negatively associated with perceived stress, whereas epistemic trust towards mother and high reflective function were negatively associated with emotional dysregulation.

Authors and Year	Country	Aim	Sample characteristics *N* (gender distribution) Age=range (*M, SD*)	Study design	Key findings
Marszał & Jańczak (2018) [51]	Poland	To verify the relationships between emotion dysregulation, mentalization and romantic attachment found in adults, in late adolescent females.	Community sample of females on last year of High-School<br><br>*N*=92<br><br>Age=18	Cross-sectional	Primitive modes of mentalizing were associated with higher levels of emotion dysregulation.<br>Adolescents with secure romantic attachment showed significantly better emotion regulation than those with insecure attachment.<br>Regression analyses indicated that attachment anxiety and mentalization significantly predicted emotion dysregulation, whereas attachment avoidance was not a significant predictor. (Anxiety being a stronger predictor than mentalization).

**Authors and Year**	**Country**	**Aim**	**Sample characteristics *N* (gender distribution) Age=range (*M*, *SD*)**	**Study design**	**Key findings**
Milesi et al. (2024) [49]	Italy		Community sample recruited from middle and high schools	Psychometric validation study	
		Study 1:<br>To examine the factorial structure of the Epistemic Trust, Mistrust and Credulity Questionnaire and assess disparities in ET, EM, EC levels between genders alongside correlations between factors and age.	Study 1:<br>*N*=662 (324 females)<br><br>Age=12-18<br>*(M=15.56, SD=2.20)*		Study 1:<br>Confirmatory Factor Analysis supported the original three-factor structure of the Epistemic Trust, Mistrust, Credulity Questionnaire, with acceptable fit indices and adequate internal consistency for all subscales.
		Study 2:<br>To explore convergent validity between ET, EM and EC and psychological constructs such as reflective functioning, emotion dysregulation and psychopathology.	Study 2:<br>*N*=417 (249 females)<br><br>Age=12-19<br>*(M=15.64, SD=2.08)*		Study 2:<br>Epistemic trust, mistrust, and credulity were significantly associated with mentalization abilities, emotion dysregulation, and general psychopathology, supporting convergent validity.<br>Gender differences: Female adolescents scored higher than males on trust, mistrust, and credulity subscales.<br>Minor age differences observed: older adolescents showed slightly higher<br>epistemic trust; no significant age differences for mistrust or credulity.

Authors and Year	Country	Aim	Sample characteristics *N* (gender distribution) Age=range (*M, SD*)	Study design	Key findings
Muzi & Pace (2022) [57]	Italy	To compare residential-care (RC), late adopted (LA) and community teenagers (COM) in the IWMs of attachment, assessed through an age-adapted semi-structured interview.<br>To compare the three groups in various domains of attachment.	Mixed sample from Residential Care, Late Adopted and Community<br><br>*N*= 117 (44% females)<br><br>Residential Care=39<br>Late Adoptees=39<br>Community=39<br><br>Age=12-19 *(M=15.5, SD=1,9)*	Cross-sectional	Attachment classifications:<br>RC adolescents showed significantly higher rates of insecure and disorganized attachment than both LA and COM adolescents.<br>LA and COM adolescents did not differ in attachment classifications.<br>Attachment-related functioning domains:<br><br>RC showed lower scores on narrative coherence, reflective functioning, secure base/safe haven (parents), social and school competence and affect regulation abilities.<br>LA showed higher hostility towards siblings than COM and no other significant difference in other attachment related domains

Authors and Year	Country	Aim	Sample characteristics<br>*N* (gender distribution)<br>Age=range (*M, SD*)	Study design	Key findings
Pace et al. (2022) [53]	Italy	To make use of an age-adapted interview (FFI) that can offer deeper associations than self-report questionnaires in domains such as reflective functioning, peer relationships and emotion regulation, surpassing the focus on attachment-only classifications.	Community adolescents recruited from High Schools<br><br>First wave:<br>*N*=772<br><br>Age=13-19<br>*(M=15.6, SD=1.2)*<br><br>Second wave:<br>Total *N*=112<br><br>*N*=56 adolescent females at risk for binge eating matched with *N*=56 peers without binge eating<br><br>Age=14-18<br>*(M=16.4, SD=1.3)*	Case control analytic study	Females at risk for binge eating showed higher rates of insecure-preoccupied attachment, lower narrative coherence, lower reflective functioning, and more anger toward mother compared with non-risk peers. Both insecure-dismissing and preoccupied attachment patterns significantly predicted higher binge-eating symptoms (explaining 15 % of variance).

Authors and Year	Country	Aim	Sample characteristics *N* (gender distribution) Age=range (*M, SD*)	Study design	Key findings
Parolin et al. (2023) [52]	Italy	To explore how three epistemic stances impact internalizing problems during preadolescence and adolescence, considering the role of mentalization and emotion dysregulation	Community sample of adolescents recruited via secondary schools, sports clubs and youth centers<br><br>N=482 (278 females)<br><br>Age=12-19 *(M=15.6, SD=2.05)*	Cross-sectional	Higher mentalization was associated with lower internalizing symptoms through reduced emotional dysregulation, supporting its role as a protective factor.<br>Epistemic trust was positively related to mentalization but not directly to internalizing problems.<br>Epistemic mistrust and credulity were connected to internalizing problems via pathways involving emotional dysregulation.
Sharp et al. (2016) [55]	USA, Texas	To examine the interplay between attachment (coherence), social cognition (hypermentalizing) and emotion dysregulation in its association with borderline features in adolescents.	Clinical sample, BPD inpatient adolescents<br><br>*N*=259 (63.1% female)<br><br>Age=12-17 *(M=15.42, SD=1.43)*	Cross sectional	Lower attachment coherence was significantly associated with higher levels of borderline features in adolescents, even after controlling for age, gender, and internalizing and externalizing symptoms. Mediation analyses showed that the association between lower attachment coherence and higher levels of borderline features was significantly mediated by hypermentalizing, whereas emotion dysregulation did not account for the relationship once hypermentalizing was included.

Authors and Year	Country	Aim	Sample characteristics *N* (gender distribution) Age=range (*M*, *SD*)	Study design	Key findings
Zhang et al. (2025) [38]	UK	To validate and assess the psychometric properties of the Adolescent Story Stem Profile (ASSP), a tool designed to assess mentalization, affect competency and attachment in adolescence	Mixed: low-risk (from secondary schools) and high-risk sample (with prior experience in foster care)<br><br>Total *N*=249 (143 female)<br><br>*N*=182 low-risk<br>*N*= 67 high-risk<br><br>Age=10-17<br>*(M=14.1, SD=1.56)*	Psychometric Validation Study	EFA concluded to three factors: Factor1: Story-self Relevance, Factor 2: Attachment, Factor 3: Mentalization and Affect competency<br>Internal consistency was satisfactory<br>Correlations of ASSP subscales were significant for the community sample<br>No significant differences between low and high-risk groups on Mentalization subscale<br>Low-risk group had better responses on Attachment subscale<br>High-risk group had higher total scores on Affective Competency subscale

Table S2. Attachment, Emotion regulation and Mentalization Measures of included studies

Authors and Year	Measures	Type	Attachment, Emotion Regulation and Mentalization Operationalization
Gambin et al. (2021) [50]	Security Scale<br><br>Reflective Function Questionnaire (RFQ)<br><br>Difficulties in Emotion Regulation Scale (DERS)	Self-report<br><br>Self-report<br><br>Self-report	Perceived attachment security to both mother and father, was expected to be associated with more adaptive emotion regulation strategies and better mentalizing abilities.<br><br>Reflective functioning was conceptualized as a mediating process linking attachment security to adolescents' access to adaptive emotion regulation strategies.
Jewell et al. (2023) [56]	Adolescents:<br>Attachment Style Questionnaire (ASQ)<br><br>Difficulties in Emotion Regulation Strategies Scale (DERS)<br><br>Hypermentalizing Questionnaire-Adolescent Version (parent scale)<br><br>Reflective Function Questionnaire-Youth (RFQY)	Self-report<br><br>Self-report<br><br>Self-report<br><br>Self-report	Attachment security was expected to be associated with forming a better therapeutic alliance at one month, and together with emotional regulation and mentalization to predict a better outcome in family-based therapy for eating disorders.<br><br>Insecure attachment, mentalizing difficulties and emotional dysregulation was expected to make family members more vulnerable in an emotionally charged situation, therefore predict poorer therapeutic alliance at one month.

Authors and Year	Measures	Type	Attachment, Emotion Regulation and Mentalization Operationalization
Jewell et al. (2023) [56] *(continued)*	Parents:<br>Reflective Function Questionnaire (RFQ8)	Self-report	An inability to consider complex models of mental states of self and others (hypomentalizing) as well as making unjustified assumptions for the mental states of others (hypermentalizing) were expected to predict therapeutic alliance and treatment outcome.
	Difficulties in Emotion Regulation Scale (DERS)	Self-report	
	Hypermentalizing Questionnaire-Parent Version (adolescent scale)	Self-report	
Jewell et al. (2024) [48]	Reflective Function Questionnaire-Youth (RFQY-5)	Self-report	Reflective functioning was operationalized using the RFQY-5 as a state-sensitive index of mentalizing, with change assessed at baseline and nine months of family-based therapy for adolescents with eating disorders.
	Difficulties in Emotion Regulation Scale (DERS)	Self-report	

Authors and Year	Measures	Type	Attachment, Emotion Regulation and Mentalization Operationalization
Liotti et al. (2023) [45]	Brief-Mentalized Affectivity Scale for Adolescents (B-MAS-A)	Self-report	Mentalized affectivity was operationalized as a mentalization-based emotion regulation capacity, reflecting adolescent's ability to identify, process and express emotions in an interpersonal context. The B-MAS-A in adolescents was used to assess dimensions of mentalized affectivity, as a transdiagnostic tool for internalizing and externalizing difficulties.
	Epistemic Trust, Mistrust, Credulity Scale Questionnaire (ETMCQ)	Self-report	
	Reflective Functioning Questionnaire (RFQ)	Self-report	
	Toronto Alexithymia Scale (TAS-20)	Self-report	
Locati et al. (2023) [54]	Reflective Functioning Questionnaire for Youth (RFQ-Y)	Self-report	Epistemic trust and mentalization were conceptualized as complementary protective mechanisms in the context of pandemic-related stress, with epistemic trust buffering perceived stress and mentalization supporting emotion regulation. These constructs were operationalized using the IPPA Trust subscale (proxy) and the RFQ-Y, respectively.
	Inventory of Parent and Peer Attachment (IPPA)	Self-report	
	Difficulties in Emotion Regulation Scale (DERS)	Self-report	

Authors and Year	Measures	Type	Attachment, Emotion Regulation and Mentalization Operationalization
Marszał & Janczak (2018) [51]	Experiences in Close Relationships Scale (ECR)	Self-report	Emotion regulation in the context of emerging romantic attachment in late adolescent females was operationalized using the DERS. Romantic attachment insecurity (anxiety and avoidance) was assessed using the ECR, and mentalization was operationalized via the MST as a capacity for processing emotional experience under conditions of affective arousal. Attachment anxiety and reduced mentalization were conceptualized as predictors of emotion dysregulation.
	Difficulties in Emotion Regulation-Scale (DERS)	Self-report	
	Mental States Task (MST)	Performance based structured task	
Milesi et al. (2024) [49]	Epistemic Trust, Mistrust, Credulity Questionnaire (ETMCQ)	Self-report	Epistemic trust, mistrust and credulity in an adolescent sample was operationalized via the ETMCQ. Construct and convergent validity were examined in relation to mentalization (RFQ-Y), emotion dysregulation (DERS), attachment-related trust (IPPA), and internalizing/externalizing difficulties (YSR).
	Reflective Functioning Questionnaire for Youth (RFQ-Y)	Self-report	
	Inventory of Parent and Peer Attachment (IPPA)	Self-report	
	Difficulties in Emotion Regulation Scale (DERS)	Self-report	

Authors and Year	Measures	Type	Attachment, Emotion Regulation and Mentalization Operationalization
Muzi & Pace (2022) [57]	Friends and Family Interview (FFI)	Semi-structured Interview	Attachment was operationalized as a multifaceted representational system, encompassing both attachment patterns and attachment-related domains such as reflective functioning, social competence and adaptive response. A sensitive narrative-based interview (FFI) was used to capture dimensional and categorical attachment-related domains within adolescent groups characterized by low, medium and high risk.
Pace et al. (2022) [53]	Friends and Family Interview (FFI)	Semi-Structured Interview	Low and medium risk binge eating was conceptualized as an emotion dysregulation and maladaptive coping in a group of community adolescents matched with healthy controls. Attachment was operationalized using a narrative-based interview (FFI) capturing attachment-related domains such as reflective functioning, affect regulation strategies and peer relationships.

Authors and Year	Measures	Type	Attachment, Emotion Regulation and Mentalization Operationalization
Parolin et al. (2023) [52]	Epistemic Trust, Mistrust, Credulity Questionnaire (ETMCQ)	Self-report	Epistemic stances (ETMCQ) were examined in relation to mentalization (RFQ-Y), emotion dysregulation (DERS), attachment-related trust (IPPA), and internalizing/externalizing difficulties (YSR) as components of an integrated protective–risk system.
	Reflective functioning questionnaire-youth (RFQ-Y)	Self-report	
	Difficulties in Emotion Regulation Scale (DERS)	Self-report	
Sharp et al. (2016) [55]	Child Attachment Interview (CAI)	Semi-structured Interview	In the context of a multiple mediational approach, hypermentalizing (MASC) and emotion dysregulation (DERS) were expected to mediate the relation between attachment security (CAI) and borderline features in adolescent inpatients.
	Measure for Assessing Social Cognition (MASC)	Performance-based, video-based assessment	
	Difficulties in Emotion Regulation Scale (DERS)	Self-report	

Authors and Year	Measures	Type	Attachment, Emotion Regulation and Mentalization Operationalization
Zhang et al. (2025) [38]	The Adolescent Story Stem Profile (ASSP)	Performance-based, narrative, semi-structured assessment tool	Attachment representations were operationalized using the ASSP, a narrative-based measure assessing attachment-related domains, mentalization, and affective competence through adolescents' responses to story stems designed to elicit affective arousal in attachment-relevant contexts.
	The reflective function questionnaire for youths (RFQY-5)	Self-report	

Note: ASQ = Attachment Style Questionnaire; ASSP = Adolescent Story Stem Profile; B-MAS-A = Brief Mentalized Affectivity Scale for Adolescents; CAI = Child Attachment Interview; DERS = Difficulties in Emotion Regulation Scale; ECR = Experiences in Close Relationships Scale; ETMCQ = Epistemic Trust, Mistrust and Credulity Questionnaire; FFI = Friends and Family Interview; IPPA = Inventory of Parent and Peer Attachment; MASC = Movie for the Assessment of Social Cognition; MST = Mental States Task; RFQ = Reflective Functioning Questionnaire; RFQ-Y = Reflective Functioning Questionnaire for Youth; RFQ-Y-5 = Reflective Functioning Questionnaire for Youth (Five-Item Version); TAS-20 = Toronto Alexithymia Scale.

Table S3. ***Basic methodological appraisal of included studies***

Study	Design	Sample type	Main measurement mode	Key strengths	Key limitations	Overall evidential contribution
Gambin et al. (2021) [50]	Cross-sectional	Community	Self-report	Large sample; examines attachment to mother and father separately; mediation model tested	Cross-sectional; adolescent self-report only; possible shared-method variance	Moderate
Jewell et al. (2023) [56]	Prospective observational cohort	Clinical	Self-report; multi-informant	Prospective design; clinical relevance; includes parent and adolescent data; treatment outcome assessed	Mostly questionnaire-based; disorder-specific sample; limited generalizability beyond eating disorders	Relatively stronger

Study	Design	Sample type	Main measurement mode	Key strengths	Key limitations	Overall evidential contribution
Jewell et al. (2024) [48]	Psychometric validation within prospective cohort	Clinical	Self-report	Clinically relevant psychometric examination; sensitivity to change assessed	Single brief self-report measure; poor psychometric performance; disorder-specific sample	Moderate for measurement critique
Liotti et al. (2023) [45]	Psychometric validation	Community	Self-report	Large samples; factor structure and convergent validity examined; multidimensional construct coverage	Community sample only; self-report only; no clinical validation	Moderate

Study	Design	Sample type	Main measurement mode	Key strengths	Key limitations	Overall evidential contribution
Locati et al. (2023) [54]	Cross-sectional	Community	Self-report	Relevant stress-context application; integrates epistemic trust and mentalization	Small sample; cross-sectional; self-report only	Preliminary to moderate
Marszał & Jańczak (2018) [51]	Cross-sectional	Community	Self-report and performance-based task	Includes performance-based mentalization task; focused relational context	Small sample; only late-adolescent females; limited generalizability	Moderate
Milesi et al. (2024) [49]	Psychometric validation	Community	Self-report	Large samples; CFA and convergent validity; useful for epistemic trust measurement	Self-report only; community sample; no longitudinal data	Moderate

Study	Design	Sample type	Main measurement mode	Key strengths	Key limitations	Overall evidential contribution
Muzi & Pace (2022) [57]	Cross-sectional	Mixed/high-risk	Semi-structured interview	Interview-based comparative design; richer attachment-related assessment; includes high-risk groups	Modest sample size; cross-sectional; limited direct measurement of emotion regulation	Moderate
Pace et al. (2022) [53]	Case-control analytic study	Community at-risk subgroup	Semi-structured interview	Interview-based assessment; matched comparison; clinically relevant eating-risk phenotype	Female-only risk group; limited generalizability; analytic subsample relatively small	Moderate

Study	Design	Sample type	Main measurement mode	Key strengths	Key limitations	Overall evidential contribution
Parolin et al. (2023) [52]	Cross-sectional	Community	Self-report	Good sample size; mediation pathways tested; integrates epistemic trust, mentalization, and dysregulation	Cross-sectional; self-report only; no clinical sample	Moderate
Sharp et al. (2016) [55]	Cross-sectional	Clinical inpatient	Interview, performance-based, and self-report	Clinical sample; multimethod design; strong construct coverage	Cross-sectional; inpatient setting may limit generalizability	Relatively stronger
Zhang et al. (2025) [38]	Psychometric validation	Mixed/high-risk	Performance-based narrative tool and self-report	Innovative narrative/performance-based tool; includes high-risk group	Early validation stage; mixed findings; limited external validation	Moderate
